# Early Emergence of CD19-Negative Human Antibody-Secreting Cells at the Plasmablast to Plasma Cell Transition

**DOI:** 10.4049/jimmunol.1501761

**Published:** 2017-05-10

**Authors:** Gururaj Arumugakani, Sophie J. Stephenson, Darren J. Newton, Andy Rawstron, Paul Emery, Gina M. Doody, Dennis McGonagle, Reuben M. Tooze

**Affiliations:** * Section of Experimental Musculoskeletal Medicine, Leeds Institute of Rheumatic and Musculoskeletal Medicine, St James’s University Hospital, Leeds LS9 7TF, United Kingdom;; † Section of Experimental Haematology, Leeds Institute of Cancer and Pathology, University of Leeds, Leeds LS9 7TF, United Kingdom; and; ‡ Haematological Malignancy Diagnostic Service, Leeds Teaching Hospitals National Health Service Trust, St James’s University Hospital, Leeds LS9 7TF, United Kingdom

## Abstract

Long-lived human plasma cells (PCs) play central roles in immunity and autoimmunity and are enriched among the subpopulation of CD19^neg^ human PCs. However, whether human CD19^neg^ PCs are necessarily aged cells that have gradually lost CD19 expression is not known. Assessing peripheral blood samples at steady-state and during the acute response to influenza vaccination in healthy donors, we identify the presence of phenotypic CD19^neg^ plasmablasts, the proliferative precursor state to mature PCs, and demonstrate by ELISPOT that these are Ab-secreting cells (ASCs). During the acute response to influenza vaccination, CD19^pos^, CD19^low^, and CD19^neg^ ASCs secrete vaccine-specific Abs and show linked *IGHV* repertoires. To address precursor/product relationships, we use in vitro models that mimic T-dependent and T-independent differentiation, finding that the CD19^neg^ state can be established at the plasmablast to PC transition, that CD19^neg^ PCs increase as a percentage of surviving PCs in vitro, and that CD19^neg^ and CD19^pos^ PCs can be maintained independently. These data provide proof-of-principle for the view that newly generated ASCs can acquire a mature PC phenotype that is accompanied by loss of CD19 expression at an early stage of differentiation and that aging is not an obligate requirement for a CD19^neg^ state to be established.

## Introduction

Long-lived plasma cells (LLPCs) have emerged as major contributors to humoral immunity (1, 2). The independence of this population of Ab-secreting cells (ASCs) from the B cell pool has profound implications for therapeutic intervention in Ab-driven immune pathologies (3). Serological analyses have suggested that the lifespan of human LLPCs may approach that of the individual (2, 4); in a recent elegant study, lifespans of decades have been inferred using carbon-14 dating approaches (5). Extended plasma cell (PC) lifespans have been demonstrated in mice (6, 7), and much interest has focused on characterizing potential survival mechanisms for such LLPCs, which may include elements intrinsic to the PC program (8–10) or related to the PC niche (11–15).

One important approach, of particular relevance to studies of human PC biology, is to identify surface phenotypes that may define underlying functional heterogeneity and link to PC longevity (16, 17). LLPCs are considered to reside principally in the bone marrow (BM), although the recent data of Landsverk et al. (5) have now also directly documented these in the small intestine. One of the major markers of phenotypic heterogeneity among human BM and intestinal PCs is the retention or loss of CD19 expression. Indeed, the absence of CD19 expression is characteristic of neoplastic PCs in early and advanced PC malignancies (18, 19), supporting the importance of distinguishing PC subpopulations based on CD19 phenotypic state.

CD19 is a coreceptor of the BCR complex and is one of the earliest and most specific markers of B-lineage commitment. In humans, the majority of PCs retain expression of CD19, whereas a minority of normal PCs lack CD19 expression (18, 20, 21). Recent studies by Mei et al. (16) and subsequently by Halliley et al. (17) indicated that BM (CD19^neg^) PCs are enriched for long-lived cells; these have been followed by the demonstration of a similar phenotypic association for small intestinal PCs (5). The direct association of CD19^neg^ PCs as a source of the long-lived anti-viral Ab response was demonstrated in an index individual in the work of Halliley et al. (17), and this association was further supported by the enrichment of vaccine-specific Abs determined by ELISPOT in the CD19^neg^ PC fraction of multiple individuals. In patients treated with anti-CD19 chimeric AgR T cell therapy, which selectively eliminates B cells and CD19-expressing PCs, the maintenance of CD19^neg^ PCs and associated vaccine/pathogen-specific IgG and IgA responses has provided further evidence for the independent maintenance of this PC fraction and accompanying long-lived immunity (22). Extending this phenotypic association beyond the BM, CD19^neg^ PCs in human small intestine were linked to virus-specific immunity and were identified as two phenotypic fractions of CD45^pos^ or CD45^neg^ CD19^neg^ PCs (5). In an elegant approach, a direct inference of average PC age was made using carbon-14 dating, which indicated a median age of 11 and 20 y for CD45^pos^ and CD45^neg^ CD19^neg^ PC populations, respectively, in the small intestine. Intriguingly, this study also identified a greater variability in the inferred average age for the CD45^pos^ CD19^neg^ PCs fraction, including an average age of 0 y for such cells in two of six individuals tested (5).

PCs are derived from B cells by a process of transcriptional reprogramming and specialization for committed Ab secretion (23). This functional specialization is coupled to extinction of many elements of the pre-existing B cell gene-expression program. The consequent transitions in surface marker expression contribute to the identification of distinct stages of B cell differentiation, including the activated B cell/preplasmablast, plasmablast (PB), and PC states (24–26). The distinction between the PB and PC states in humans is closely linked to acquisition of surface CD138 expression, but it is strictly defined by entry into cell cycle quiescence (27). Therefore, the encompassing term ASCs is used to include the PB and PC states when the proliferative state is not defined. How CD19^pos^ and CD19^neg^ PCs are generated in humans is not known, and two distinct models can be envisaged. The first is that CD19^pos^ PCs gradually lose CD19 expression accompanying prolonged ageing in an appropriate niche environment; absence of CD19 would then provide a marker of PC age. The second model is that loss or retention of CD19 may be established during the initial phase of commitment to the PC state, with subsequent recruitment into the long-lived pool. Indeed, these two models are not necessarily mutually exclusive, but the second model would imply that absence of CD19 expression does not necessarily identify an individual PC or group of PCs as aged. In this article, we address whether CD19^neg^ ASCs emerge early during PC differentiation in vivo in the response to influenza vaccination, as well as in the context of in vitro model systems that allow the sequential tracking of cell states in a differentiating human ASC population, providing proof of principle for early emergence of CD19^neg^ ASCs in vitro and in vivo.

## Materials and Methods

### Samples and cell isolation

Approval for this study was provided by the U.K. National Research Ethics Service via the Leeds East Research Ethics Committee (approval reference: 07/Q1206/47). The use of surplus clinical samples from anonymous patients and controls was approved by the Leeds Teaching Hospitals Research Ethics Committee (approval reference: 04/Q1107/40).

Peripheral blood was obtained from healthy donors after informed consent. Samples from healthy volunteers selected from within the academic environment, without evidence of recent infection or other clinical condition, undergoing routine annual seasonal influenza vaccine were collected at the specified time points. Sequential sampling for time-course experiments derived from seasonal influenza vaccination in 2014, whereas ELISPOT and *IGHV* sequence analysis derived from seasonal influenza vaccination in 2015. All vaccines were delivered as part of routine vaccination programs for National Health Service and University staff, with ELISPOT and *IGHV* sequences relating to vaccination with inactivated influenza vaccine (Split Virion) BP 2015/2016 strain. Donors included were between 25 and 50 y of age, and BM aspirates were from anonymous donors and were derived from surplus clinical samples assessed as normal by clinical flow cytometry. RBCs were lysed (ammonium chloride 8.6 g/l) before flow cytometric analysis. Mononuclear cells were isolated by Lymphoprep (Alere) density gradient centrifugation. Total B cells were isolated by negative selection with a Memory B Cell Isolation Kit, according to the manufacturer’s instructions (Miltenyi Biotec).

### Abs and reagents

Abs used were CD19 PE (LT19; Miltenyi Biotec), CD138 allophycocyanin (B-B4; Miltenyi Biotec), CD38 PE-Cy7 (HB7; BD Biosciences), CD38 AF700 (HIT2; BioLegend), CD20 eFluor V450 (2H7; eBioscience); CD27 AF647 (LT27; AbD Serotec), CD27 FITC (M-T271), CD19 PerCP-Cy5.5 (SJ225C1; BD Biosciences), CD19 PE-Cy7 (SJ225C1; BD Biosciences), CD24 FITC (ML5; BD Biosciences), CD84 PE (CD84.1.21; BioLegend), CD38 PerCP-Cy5.5 (HIT2; BD Biosciences), CD95 BV421 (DX2; BioLegend), CD20 allophycocyanin-H7 (L27; BD Biosciences), CD27 BV605 (O323; BioLegend), CD3 VioGreen (BW264/56; Miltenyi Biotec), Ki67 FITC (B56; BD Biosciences), unconjugated goat anti-IRF4 (M-17; Santa Cruz Biotechnology), and donkey anti-goat IgG AF488 (polyclonal; Invitrogen). Controls were isotype-matched mouse mAbs. Annexin V FITC was from eBioscience, and 7-AAD was from BD Biosciences.

Reagents included human IL-2 (Roche), IL-6 (PeproTech), IFN-α (Sigma), IL-21 (PeproTech), goat anti-human F(ab′)_2_ fragments (anti-IgM and anti-IgG; Jackson ImmunoResearch), HybridoMax hybridoma growth supplement (Gentaur), Lipid Mixture 1, Chemically Defined (200×), and MEM Amino Acids Solution (50×, Sigma).

### Flow cytometry and cell sorting

Cells were analyzed using direct immunofluorescence staining on a BD LSR II Fortessa flow cytometer (BD Biosciences). Cell populations were gated on forward scatter and side scatter profiles for viable cells, and costaining with Annexin V FITC and 7-AAD was also used to exclude dead cells for in vitro experiments. Analysis was performed with BD FACSDiva Software 8.0 (BD Biosciences). For in vitro experiments, cells were sorted based on CD19 or CD27 expression with MoFlo high-speed cell sorter (Beckman Coulter) or a BD Biosciences Influx, at the indicated time points. Graphic overlays were generated with FlowJo Vx, and the rest of the flow cytometry plots were generated with FACSDiva V8.0. Statistical analysis was performed with GraphPad Prism 6.

Absolute cell counts were performed with CountBright beads (Invitrogen) for in vitro experiments. Dual-platform methodology, with cell counts from Sysmex K-1000, was used for peripheral blood B cell counts. Intracellular staining was performed with a BD IntraSure Kit (BD Biosciences).

**FIGURE 1.**
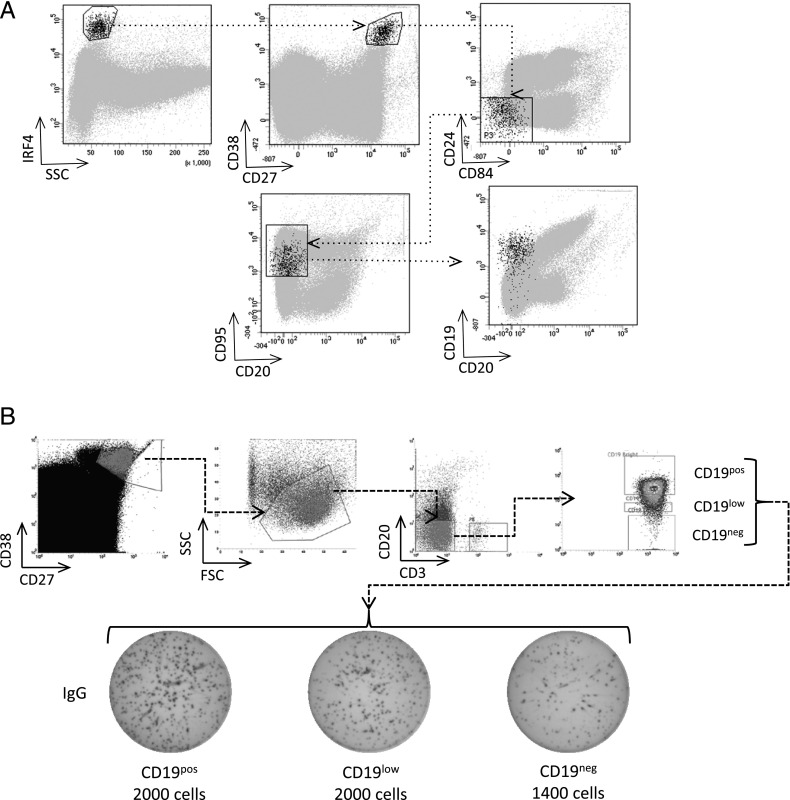
CD19^neg^ PBs are a functional ASC population in the peripheral blood. (**A**) PBs were gated based on their scatter profile, intracellular expression of IRF4, strong surface expression of CD27 and CD38, and negativity for CD24 and CD84. The expression of CD20 and CD19 within the phenotypically defined PBs is shown in black against a background of other events in gray. Plots are representative of samples from three healthy volunteers. (**B**) PBs (CD38^high^, CD27^high^, and CD20^neg^) were flow sorted into three fractions based on the expression level of CD19 (pos, low, neg) and assessed by ELISPOT for the capacity to secrete IgG. The number of cells seeded in each well is indicated (a representative example of two independent experiments is shown).

Peripheral blood was obtained from healthy donors at 6–8 d (influenza-specific response) or 67+ d postvaccination (baseline). Mononuclear cells were isolated by Lymphoprep (Alere) density gradient centrifugation. T cells were depleted with CD3 magnetic beads (Miltenyi Biotec) prior to cell sorting on a BD Influx (BD Biosciences). ASCs were then sorted on their forward/side scatter profile and surface expression of CD27^high^/CD38^high^/CD3^−^/CD20^−^. These cells were then further separated into CD19^pos^, CD19^low^, and CD19^neg^ populations, as depicted in Fig. 1.

### ELISPOT

For detection of influenza-specific Ig, polyvinylidene difluoride plates were activated with 70% ethanol, washed with sterile water, and coated with human influenza vaccine (2.25 μg per well in sterile PBS) overnight at 4°C. Plates were then washed with sterile PBS and blocked with IMDM containing 10% FBS, IL-21 (100 ng/ml), and IL-6 (20 ng/ml). Cells were added at the indicated number in IMDM containing 10% FBS. Plates were then incubated for 18–24 h in a humidified incubator. Following incubation, cells were removed, and plates were washed with PBS. To detect influenza-specific IgG secretion, plates were incubated with IgG–MT78–Biotin (1 μg/ml in PBS/0.5% BSA) for 2 h, washed with PBS, and incubated with Streptavidin-HRP for an additional hour. Plates were washed, and spots were detected by addition of TMB substrate solution. Images were captured using an AID EliSpot Reader System with software version 5.0 (Autoimmun Diagnostika).

Detection of typhoid-specific Ig was carried out in a similar manner, with the exception that plates were coated with Purified Vi Capsular Polysaccharide of *Salmonella enterica*, serovar Typhi (1.5 μg per well in sterile PBS).

Vaccines used for coating plates were inactivated Influenza vaccine (Split Virion) BP 2015/2016 strain and Typhim Vi Purified Vi Capsular Polysaccharide of *Salmonella* Typhi (Ty 2 Strain), both manufactured by Sanofi Pasteur MSD.

For detection of human IgG secretion, a Human ELISpot^PLUS^ IgG kit (Mabtech) was used. The assay was performed as described in the manufacturer’s protocol, and ASCs were added as indicated in the figures.

### *IGHV* sequencing and analysis

For analysis of the Ig repertoire, *IGHV* regions were amplified from randomly primed cDNA made from the flow-sorted CD19^pos^, CD19^low^, and CD19^neg^ PB fractions using tagged framework-1 BIOMED-2 primers and proofreading Phusion polymerase to minimize amplification errors (28). Following second-round indexing with Nextera NGS tags (Illumina), libraries were sequenced using MiSEquation 2 × 300-bp paired-end reads. Resultant sequences were overlapped using FLASH (fast length adjustment of short reads) (29) and uploaded to the Galaxy Web-based platform (http://usegalaxy.org) for quality control and further analysis. These sequences were then groomed, trimmed, filtered to remove low-quality sequences (99% of sequence quality score > 20), and output as FASTA files (30). The sequences were analyzed with HighV-QUEST (31); because of the large number of sequences generated and limitations on IMGT submissions, the FASTA files were split into individual files of <500,000 reads, and the results were joined back together using custom Python scripts. Nonproductive rearrangements and unknown rearrangements were removed from the analysis, and the initial cell number was used to determine the sequencing depth required per cell (reporting at a ratio of 0.5 reads per cell).

For comparisons, the 100 most frequent *IGHV* rearrangements from each cell fraction were considered. Overlaps between *IGHV* rearrangements between populations were restricted to sequences encoding identical CDR3 amino acid sequences, including identical CDR3 lengths, with identical V and J family sequences (32). This restrictive approach excluded rare events of identical CDR3 sequences associated with nonidentical underlying V and J family sequence, as well as polymerase errors. Furthermore, any bias in this approach tends to underestimate clonal lineage relationships between populations, by not considering relationships between CDR3 sequences that differ as a result of somatic hypermutation. To assess the extent of population overlap, Morisita–Horn (MH) indices (33) were calculated in Excel for the top 100 *IGHV* rearrangements, defined as above. Violin plots of somatic hypermutation distribution were generated with ggplot2 in R. The overlap of the most prevalent clones in each sample was displayed using Circos plots (34).

### In vitro differentiation

Twenty-four–well flat-bottom culture plates (Corning) and IMDM supplemented with GlutaMAX and 10% heat-inactivated FBS (Invitrogen) were used, unless otherwise specified.

### Days 0–3

B cells were cultured at 2.5 × 10^5^ per milliliter with IL-2 (20 U/ml), IL-21 (50 ng/ml), and F(ab′)_2_ goat anti-human IgM and IgG (10 μg/ml) on gamma irradiated CD40L-expressing L cells (6.25 × 10^4^ per well). R848 (1 μg/ml) was used instead of CD40L where specified.

### Days 3–6

At day 3, cells were detached from the CD40L L cell layer and reseeded at 1 × 10^5^ per milliliter in media supplemented with IL-2 (20 U/ml), IL-21 (50 ng/ml), HybridoMax hybridoma growth supplement (11 μl/ml), Lipid Mixture 1, Chemically Defined, and MEM Amino Acids Solution (both at 1× final concentration).

### Day 6 onward

For extended lifespan maintenance, day-6 cells were harvested and recultured, at 3 × 10^5^ cells per well (total volume 2.1 ml), in the upper compartment of a 12-well Transwell insert (clear polyester membrane, 0.4-μm pore; Corning). Gamma irradiated M2-10B4 stromal cells were seeded into the lower chamber, at 8.32 × 10^4^ per well, proportionally. Cells were grown in IMDM supplemented with IL-6 (10 ng/ml), IL-21 (50 ng/ml), IFN-α (100 U/ml), HybridoMax hybridoma growth supplement (11 μl/ml), Lipid Mixture 1, Chemically Defined, and MEM Amino Acids Solution. IL-21 was discontinued after day 13. Every 3.5 d, one half of the media volume was exchanged. A detailed protocol is available on request.

## Results

### CD19^neg^ PBs are present in the peripheral blood

LLPCs are enriched among CD19^neg^ BM PCs and are thought to derive from homing and residency of peripherally generated PBs/PCs to the BM. It is not known whether CD19 loss is a gradual process that is directly linked to PC age or whether it may be established during the initial phase of ASC generation. To address this question, we began by analyzing peripheral blood before and after seasonal influenza vaccination in healthy donors. The peripheral blood contains populations of ASCs, which can be phenotypically defined and are thought to predominantly represent recently generated PBs. However, more mature pre-existing PCs displaced from niche environments in the context of immune responses may also be present among peripheral blood ASCs. Using the gating strategy shown (Fig. 1A), we found a heterogeneous pattern of CD19 expression among the SSC^low^, IRF4^high^, CD27^high^, CD38^high^, CD20^neg^, CD95^pos^ peripheral blood cells, including a fraction that was CD19^neg^. To verify that the phenotypically defined populations included functional ASCs we sorted CD20^neg^, CD3^neg^, CD38^high^, CD27^high^ cells into three fractions based on CD19 expression levels (CD19^pos^, CD19^low^, and CD19^neg^) and performed ELISPOT analysis focusing on IgG secretion (Fig. 1B). This demonstrated that all three sorted populations included IgG-secreting cells. The observed number of IgG spots as a proportion of input cells appeared modestly reduced in the CD19^neg^ fraction, potentially consistent with a contribution from displaced BM PCs with compromised function, but nonetheless demonstrating that the CD3^neg^, CD19^neg^, CD20^neg^, CD27^high^, CD38^high^ peripheral blood fraction included functional ASCs.

### Ag-specific CD19^neg^ PBs are generated during acute immune responses in humans

**FIGURE 2.**
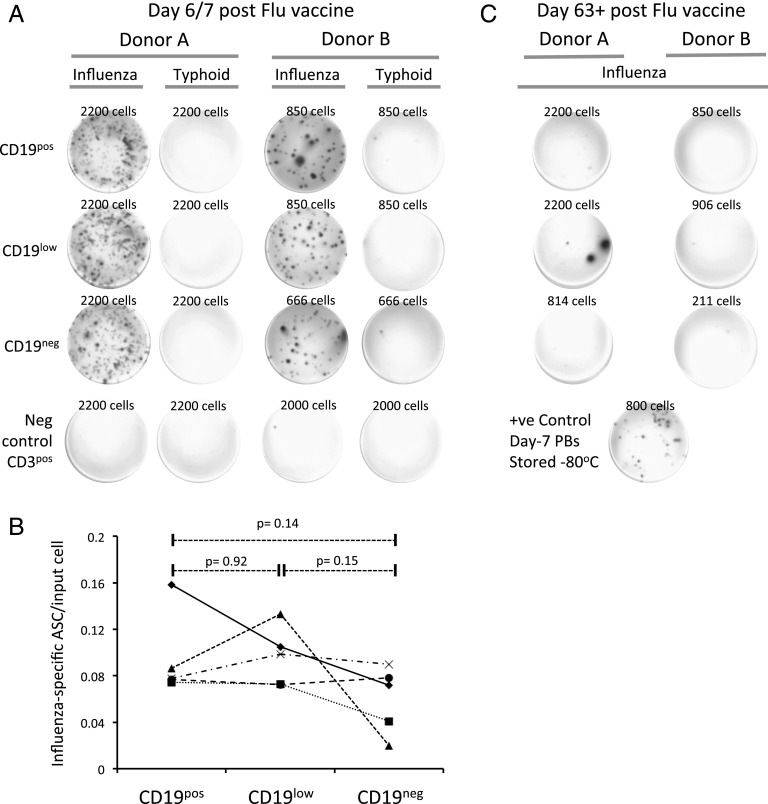
CD19^neg^ PBs generated following influenza vaccination include vaccine-specific ASCs. (**A**) ELISPOT results for two representative donors are shown at peak of the postvaccine immune response (days 6/7) and after 63+ d. For each donor, PBs were sorted into CD19^pos^, CD19^low^, and CD19^neg^ fractions, as shown in Fig. 1B, and seeded at the densities shown (determined by PB recovery). ELISPOTs show detection with anti-IgG for plates coated with immunizing influenza vaccine or with typhoid vaccine as negative control. During the peak immune response, the sorted CD3^pos^ fraction was used as an additional negative control. (**B**) A summary of influenza-specific ELISPOT results obtained from PB fractions of five independent donors is shown. Each donor is shown as a different symbol, with lines connecting the respective results for each PB fraction as indicated on the *x*-axis. The *y*-axis shows the fraction of influenza-specific ASCs detected as a proportion of input cells. Significance was calculated using a paired two-tailed *t* test. (**C**) ELISPOT results are shown for donors as in (A), with samples taken at day 63+ after vaccination. Cell numbers used in each condition are indicated. A positive control was provided by PBs derived at peak immune response and stored at −80°C.

We next took advantage of seasonal influenza vaccination to ask whether CD19^neg^ PBs were generated as Ag-specific cells during acute immune responses in human. To directly assess whether CD19^neg^ PBs included Ag-specific cells rather than simply displaced BM PCs, we sorted the CD19^pos^, CD19^low^, and CD19^neg^ PB fractions from five donors at day 6 or 7 postinfluenza vaccination and used these cells in ELISPOT assays against the vaccination Ag or against typhoid vaccine as an unrelated Ag. The phenotypic PB fractions could be recovered from all donors, albeit at differing frequencies. As expected, vaccine-specific IgG and IgM responses were readily detectable from CD19^pos^ PBs (Fig. 2A, data not shown). Strikingly, in all donors, vaccine-specific IgG Ab responses were also readily detectable from CD19^low^ and CD19^neg^ PBs (Fig. 2A). Cell numbers precluded parallel assessment of other isotype secretion from CD19^neg^ PBs. In the postvaccine response, there was no statistically significant difference in the frequency of influenza vaccine-specific ASCs (as determined by individual ELISPOTs) per input cell among any of the three fractions (Fig. 2B). To further assess the specificity of the detected PB response, donors were resampled after a 9-wk interval, at which point the acute Ag-specific immune response would be expected to have concluded. Indeed, after this interval, influenza vaccine–specific Ab secretion was markedly reduced among the peripheral blood PB fractions (Fig. 2C). Therefore, we conclude that Ag-specific CD19^neg^ PBs are generated during the acute influenza vaccine response in humans.

### Kinetics of CD19^low/neg^ PBs after influenza vaccination

**FIGURE 3.**
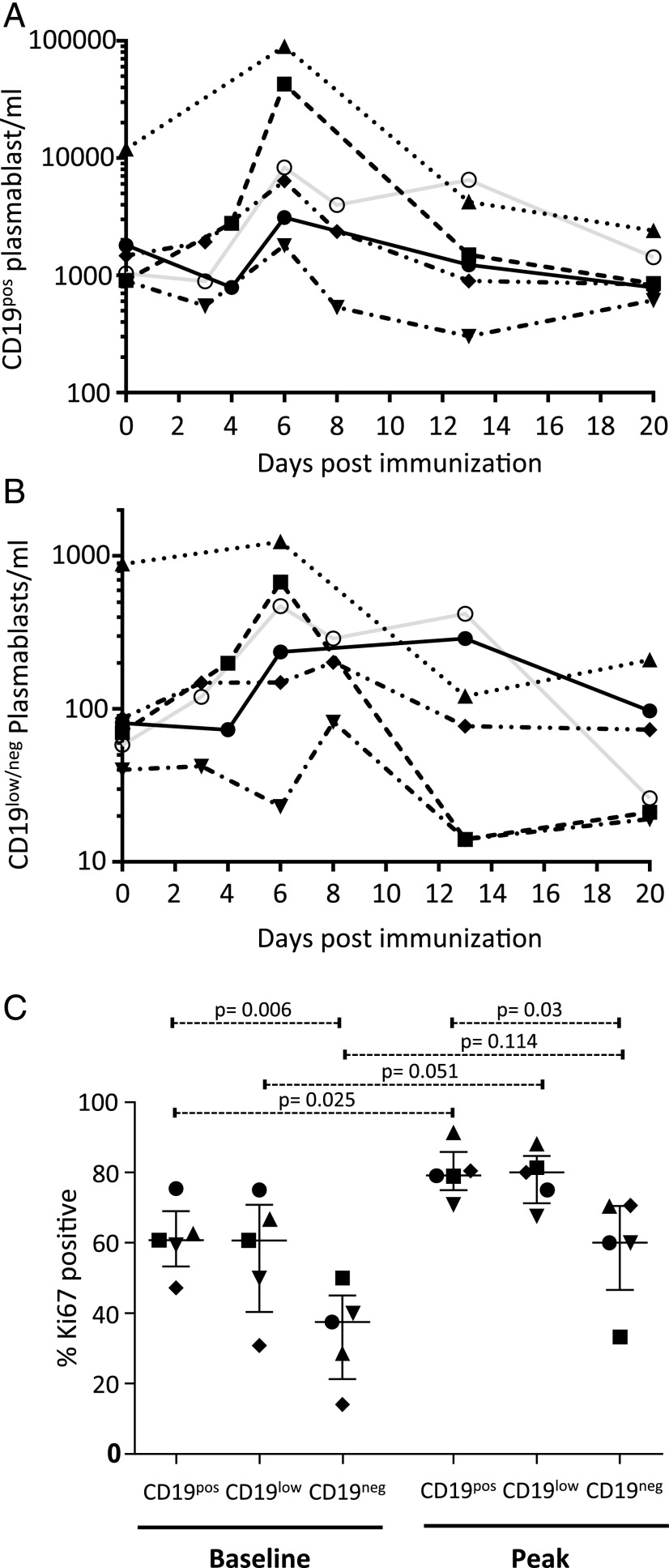
CD19^neg^ PBs increase following influenza vaccination but represent a minor fraction of peripheral blood PBs. The peripheral blood numbers per milliliter of CD19^pos^ PBs (**A**) and number of CD19^neg^ PBs (**B**) from six healthy volunteers who underwent seasonal influenza vaccination are shown at the specified time points after vaccination (baseline immediate prevaccination is *t* = 0). Individual donors are identified by the same symbols and lines in each graph (gating strategies for cell enumeration are shown in Supplemental Fig. 1A). (**C**) The percentage of Ki67^pos^ PBs in the CD19^pos^, CD19^low^, or CD19^neg^ fractions from five individual donors are shown (gating strategies for Ki67 detection are shown in Supplemental Fig. 1B) at baseline and at peak PB response (day 6). Significance was determined using a paired two-tailed *t* test.

We next analyzed the kinetic changes in peripheral blood PB populations following vaccination. Because of the continuous nature of the CD19 expression distribution and the scarcity of the CD19^neg^ fraction for these analyses, the CD19^low^ and CD19^neg^ PB fractions were considered together (Supplemental Fig. 1A). The overall PB response followed the expected course, with a substantial expansion in most donors within 6 d, followed by a subsequent contraction with variable kinetics (Fig. 3A). Of note, one donor showed a high baseline peripheral blood PB number, which ultimately returned to lower levels on subsequent sampling. Although there were no self-reported symptoms of infection or inflammatory process, we interpret the high baseline PBs in this donor, with the eventual return to PB levels similar to other donors, as representing evidence of an active immune response at the time of initial sampling prior to immunization. CD19^pos^ PBs represented the major population at baseline, accounting for >92% of PBs in all donors. Although CD19^low/neg^ PBs did respond numerically following immunization (Fig. 3B), this response was of a lower magnitude than the corresponding increase in CD19^pos^ PBs, leading to a reduction in the percentage of CD19^low/neg^ PBs in the peripheral blood from a median of 5% at baseline to 2% at day 6, with variable recovery toward steady-state levels by day 20. In two donors, CD19^neg^ PBs showed an extended numerical response, with maintained absolute numbers at days 6 and 13 of sampling. This was mirrored in one of the two donors by a similarly extended response in total PB numbers at day 13. Although the significance of this delayed kinetics is uncertain, a similar variation in PB kinetics has been observed in tetanus toxoid–specific immune responses (35). In all donors, the peak of the absolute PB response occurred at day 6, consistent with previous reports. Furthermore, consistent with a direct involvement in the Ag-specific immune response, the percentage of Ki67^pos^ PBs increased across CD19^pos^, CD19^low^, and CD19^neg^ PBs (Fig. 3C, Supplemental Fig. 1B). Thus, in the context of an acute in vivo immune response to vaccination, CD19^pos^ PBs represent the numerically predominant responding population, although CD19^low^ and CD19^neg^ PBs are present at the steady-state and during an acute immune response in human peripheral blood.

### CD19^pos^, CD19^low^, and CD19^neg^ PBs express related *IGHV* CDR3 sequences during the influenza vaccine immune responses

**FIGURE 4.**
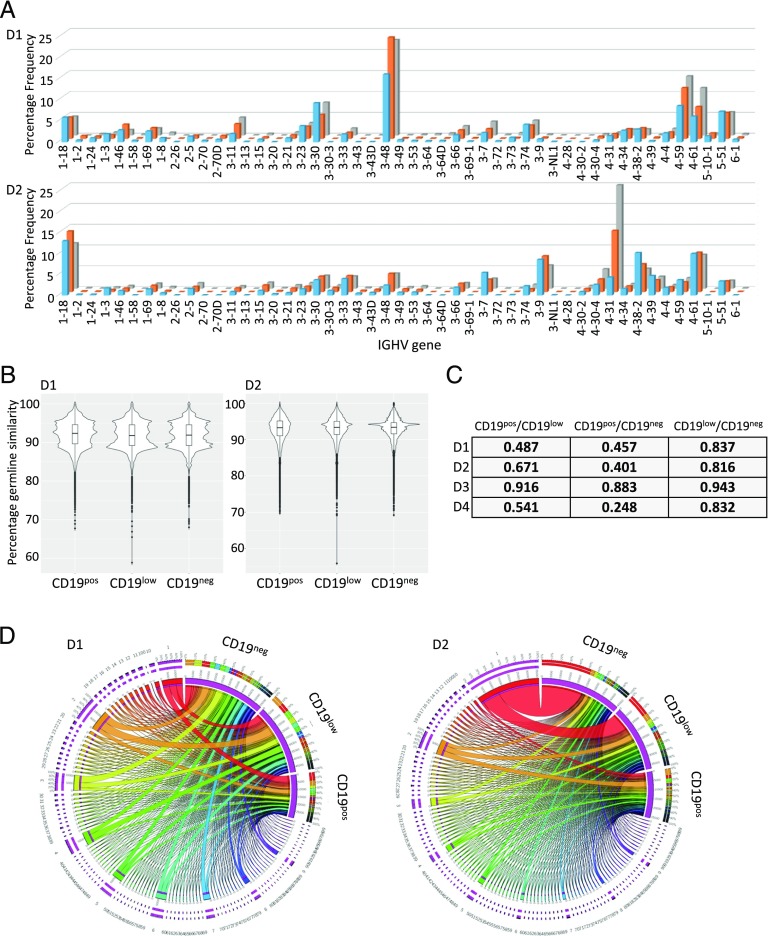
Clonal relationships of CD19^pos^, CD19^low^, and CD19^neg^ PBs following influenza vaccination. Peripheral blood from four donors, following seasonal influenza vaccination, was sorted into CD19^pos^, CD19^low^, and CD19^neg^ PBs at peak immune response using the sorting strategy shown in Fig. 1B. *IGHV* HCDR3 repertoire analysis was performed by next-generation sequencing, and analysis was performed using IMGT (see also Supplemental Fig. 2). (**A**) Shown is the distribution of *IGHV V-gene* family usage across the three PB fractions in two independent donors. (**B**) The distribution of somatic hypermutation load across the top 100 unique clones for the three PB fractions is shown from the same two donors (*y*-axis shows the percentage similarity to germline sequence, where ≥98% is considered unmutated). (**C**) MH indices are shown for the degree of population overlap based on the top 100 unique clones for the three PB fractions from each donor. (**D**) The clonal relationships for the top 100 clones are illustrated using Circos plots. Overlap of clones between PB fractions was defined based on *IGHV* rearrangements encoding identical HCDR3 sequences of the same lengths using the identical V- and J-gene segments. Values used in generating the plots were the percentage of sequence reads for each clone across all reads for the indicated cell type. The three PB fractions are separated and indicated in the upper right of each plot. The clonal relationships are linked by joining ribbons, with the clonal association indicated by color code, and at the common points of origin around the circumference of the plot. The widths of the ribbons reflect the percentage of sequence reads for the indicated clone within each PB fraction.

To further assess relationships among the peripheral blood PB populations, we sorted CD19^pos^, CD19^low^, and CD19^neg^ PBs from four additional donors at day 6 after influenza vaccination and performed repertoire sequencing of expressed *IGHV* rearrangements from mRNA. Given the variation in population frequencies, a different absolute number of input cells was recovered and sequenced for each population and donor. Across all samples, we obtained ≥640,000 high-quality paired end-reads, leading to a predicted read depth of ≥25 reads per cell (Supplemental Table I). As expected, a wide range of unique *IGHV* sequences was recovered from the three phenotypic PB fractions across all donors. This reflected, in part, the differences in overall population frequency reflected in the cell number obtained from sorting. Individual donors showed consistency across the pattern of *IGHV* segment usage (Fig. 4A, Supplemental Fig. 2C) and the ratio of unique *IGHV* sequences recovered per input cell across the three PB fractions analyzed (0.33–0.35, 0.20–0.36, 0.63–0.74, and 0.42–0.70 for donors 1–4; Supplemental Table I). In each cell fraction, and for each donor, some *IGHV* sequences were observed at high frequency within a background of rare *IGHV* rearrangements, particularly in the CD19^pos^ fraction. In terms of *IGHV* gene segment usage, the three PB fractions showed highly related overall patterns in each donor (Fig. 4A, Supplemental Fig. 2C). Consistent with derivation from an ongoing T-dependent immune response, the majority of *IGHV* sequences showed evidence of somatic hypermutation, with a very similar average and distribution of somatic hypermutation load (Fig. 4B, Supplemental Fig. 2A, 2D). Using a cut-off of <2% sequence divergence to identify unmutated *IGHV* sequences, there was no significant difference in the frequency of unmutated *IGHV* sequences in the three populations (2.3–7.1, 2.3–4.8, and 2.0–5.2% of sequences in the CD19^pos^, CD19^low^, and CD19^neg^ fraction, respectively, Supplemental Fig. 2B).

To address relationships among the phenotypic PB fractions, we focused on the 100 most prevalent HCDR3 sequences obtained from each of the three PB fractions. We considered overlaps among the populations in a restrictive fashion as *IGHV* rearrangements using identical *IGHV* and *IGHJ* gene segments and encoding HCDR3s of identical amino acid sequences and length. By limiting our assessment in this fashion, any bias favors an underestimate of relationships among the PB fractions. MH indices, which provide a statistical measure of population dispersion (MH index = 0, no overlap in terms of species/clones; MH index = 1, species/clones occur in the same proportion in both samples), supported a substantial degree of overlap among samples (Fig. 4C). Although highest for the comparison between CD19^low^ and CD19^neg^ fractions (MH indices 0.816–0.943), the degree of overlap also remained substantial in the comparison between CD19^pos^ and either CD19^low^ or CD19^neg^ fractions for all four donors (MH indices 0.487–0.916 and 0.248–0.883, respectively). We used Circos plots to visually display the overlap in *IGHV* rearrangements among populations (Fig. 4D, Supplemental Fig. 2E). These plots illustrated the common contribution that shared *IGHV* rearrangements encoding identical HCDR3 sequences made to the CD19^pos^, CD19^low^, and CD19^neg^ PB fractions in each donor. Most strikingly, in one donor (donor 2), a single recurrent *IGHV* sequence accounted for 1.4, 10, and 24% of sequence reads in the CD19^pos^, CD19^low^, and CD19^neg^ fractions, respectively (Fig. 4D); interestingly, this donor had no previous history of influenza vaccination. Overall, this analysis demonstrated that, during the acute response to influenza vaccination in humans, CD19^neg^ PBs are generated within the first week following vaccination that share *IGHV* sequences encoding identical HCDR3 sequences to those of PBs that retain CD19 expression. Because the HCDR3 sequence is a primary contributor to Ag specificity (36), and set alongside the data on secretion of vaccine-specific Abs shown in Fig. 2, we conclude that generation of Ag-specific CD19^neg^ PBs is part of the in vivo immune response to vaccination in humans.

### CD19^neg^ PCs are generated at the PB to PC transition in vitro

The in vivo analysis provides evidence for early generation of CD19^neg^ ASCs with the phenotypic characteristics of PBs during Ag-specific immune responses. However, it is not possible to track a precursor/product relationship with BM CD19^neg^ PCs in these analyses, and exploring in vivo ASC dynamics in humans represents a major challenge. To address the potential relationship between early loss of CD19 and the generation of CD19^neg^ PCs in human cells, we turned to in vitro models of long-lived PC differentiation. We (26) and other investigators (37) have described models that allow the generation and maintenance of long-lived human PCs in vitro. Both in the work of Jourdan et al. (37) and in our model system for generation of human long-lived PCs, the PCs generated in vitro were identified as CD19^low^. However we noted in subsequent differentiations that this was not uniformly the case and that subpopulations of CD19^neg^ PCs were generated.

**FIGURE 5.**
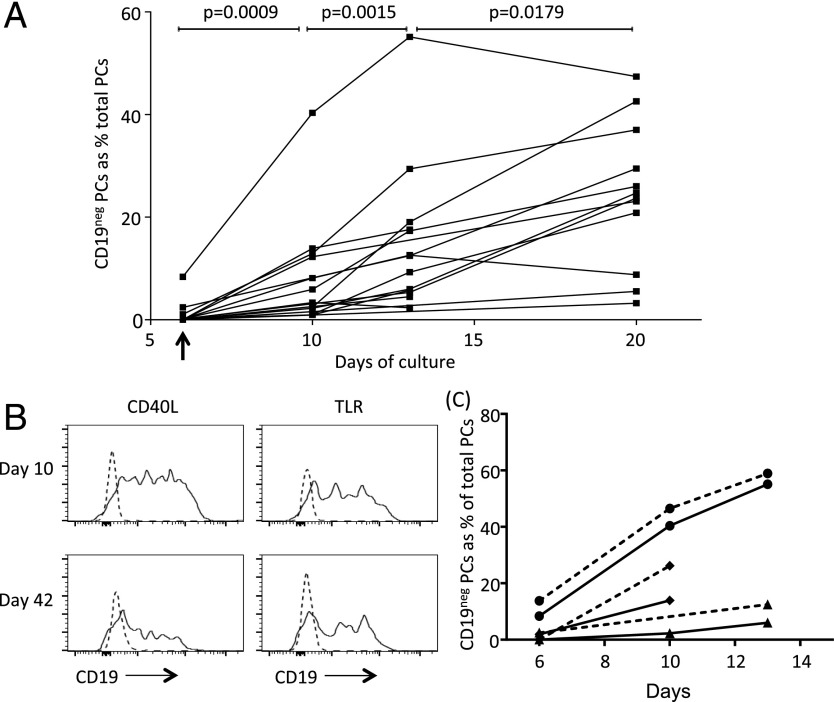
CD19^neg^ PCs are generated during the PB to PC transition in vitro. (**A**) The proportion of CD19^neg^ PCs among total PCs is illustrated in the line graph at the specified time points during in vitro differentiation. The day-6 point (when PBs were transferred into PC conditions) is indicated by an arrow on the *x*-axis. PCs were defined as Annexin-V, 7-AAD, CD20 negative, and CD38, CD138 positive. Two-tailed paired *t* test values are shown for comparison between time points. (**B**) Graphs show levels of CD19 expression by PCs generated in vitro with CD40L (left panels) or TLR stimulation (right panels) compared with isotype controls (dashed line) on days 10 and 42. (**C**) Proportion of CD19^neg^ PCs among total PCs generated in vitro using CD40L (solid lines) or TLR (dashed lines) stimulation at the specified time points in donor 1 (●), donor 2 (▴), and donor 3 (♦). Data are representative of three donors used in two different experiments.

Differentiation of peripheral blood B cells to the PB state follows a well-defined pattern leading to the generation of CD19^pos^ PBs from CD19^pos^ activated B cells (Supplemental Fig. 3A). We previously showed that transfer of such PBs into conditions promoting PC survival is accompanied by a cessation of cell proliferation within 4 d of culture (day 10). This is further supported by our gene-expression analyses demonstrating extinction of cell cycle and cell proliferation signatures over a similar timeframe (26, 38). The change in proliferative capacity represents the primary functional criterion for the transition from PBs to PCs (27) and correlates with the population level acquisition of CD138 expression. Therefore, we examined the transition in the CD19 expression state between the day 6 and day 10 time points of the in vitro culture. Cells with very low/absent CD19 expression were established rapidly among maturing PBs; they were evident as a very small proportion of CD138^pos^ cells at day 6 of culture, but they made up a substantially greater fraction of the CD138^pos^ population by day 10. Indeed, the levels of CD19 expression observed among in vitro–generated PCs (Supplemental Fig. 3B) mirrored those observed among primary BM-derived PCs (Supplemental Fig. 3C). As a percentage of PCs, the contribution of CD19^neg^ PCs varied from 1 to 44% across a range of donors at day 10. On continued culture in most donors, the percentage of CD19^neg^ PCs increased, reaching a range of 9–47% (median 25%, 95% confidence interval 21–42%) by day 20 of in vitro culture (5) (Fig. 5A). The increase in CD19^neg^ PCs was statistically significant at each successive time point of comparison, but it was most significant during the initial transition from PB to PC (day 6 versus 10, *p* = 0.0009; day 10 versus 13, *p* = 0.0015; day 13 versus 20, *p* = 0.0179; paired *t* test). Thus, CD19^neg^ PCs are generated in vitro at early time points corresponding to the point of functional and phenotypic transition to the PC state.

### TLR-dependent B cell activation, mimicking a T-independent response, generates CD19^neg^ PCs in vitro

Models of PC differentiation can be broadly divided into those using mimics of a T-dependent immune response utilizing CD40L and those modeling T-independent immune responses via the TLR pathway. Our existing model used CD40L as a principal signal to drive PC differentiation. To assess whether emergence of CD19^neg^ PCs was differentially controlled depending on the mode of B cell activation, we assessed the capacity of TLR ligation, as a mimic of a T-independent immune response, to drive differentiation of LLPCs in vitro. We found that a TLR7/8 agonist, in conjunction with IL-21 and IL-2, efficiently generated populations of PBs with a similar phenotypic transition to those generated by CD40L-based differentiation (Supplemental Fig. 3D). On subsequent transfer into the PC-maturation conditions, previously characterized as suitable for generation of LLPCs from CD40:CD40L-derived PBs, maturation to CD138^pos^ PCs and PC survival were observed for PBs generated using TLR agonists (Supplemental Fig. 3E). Thus, a differentiation system based on a synthetic TLR7/8 agonist generated PBs that were competent for maturation into LLPCs in vitro. Furthermore, PCs generated in response to a TLR7/8 agonist showed a similar pattern of heterogeneous CD19 expression from early time points (Fig. 5B), and the percentage of CD19^neg^ PCs present increased with time of culture (Fig. 5C). Thus, CD19^neg^ PCs can be generated by stimuli mimicking T-dependent (CD40) or T-independent (TLR) immune responses.

### CD19^neg^ PCs increase over time in vitro

**FIGURE 6.**
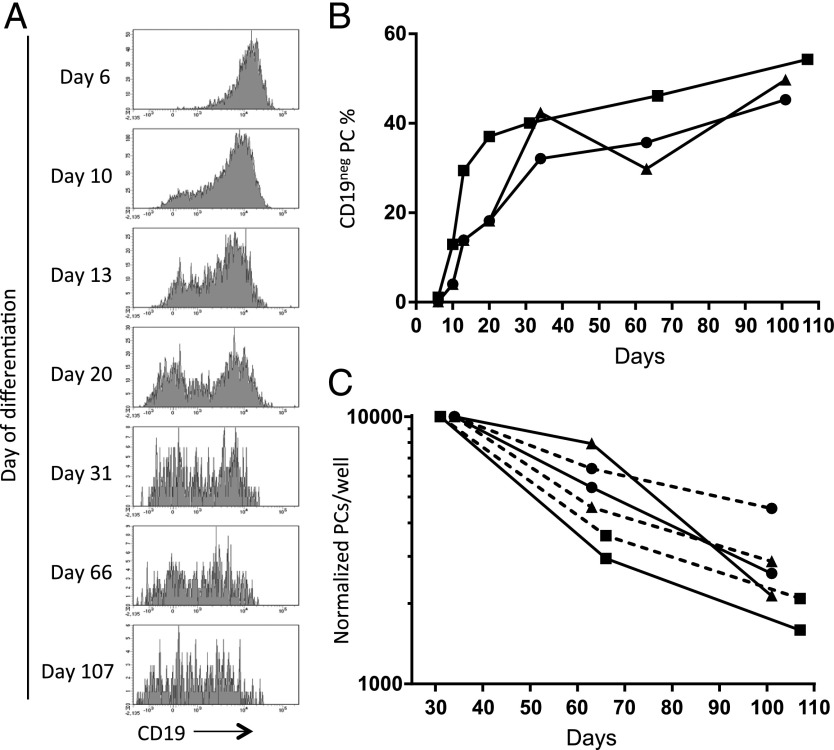
The proportion of CD19^neg^ PCs increases with time during extended in vitro culture. (**A**) Graphs showing changes in CD19 expression among in vitro–generated PCs (defined as CD20^neg^ CD38^pos^ CD138^pos^) during culture to day 107. Data shown are representative of three replicates over two experiments. (**B**) CD19^neg^ PCs generated in vitro from day-6 unsorted PBs or sorted on day 6 based on CD27 expression are shown as a proportion of total PCs during extended cultures. Data derive from independent experiments with unsorted (▪) or sorted PBs (CD27^high^ [▴]; CD27^low^ [●]). (**C**) Normalized numbers of CD19^pos^ and CD19^neg^ PCs detected per well shown at the specified time points of extended in vitro culture data derive from the same differentiations as in (**B**). CD19^pos^ PCs (solid lines) and CD19^neg^ PCs (dashed lines) derived from unsorted PBs (▪), sorted CD27^high^ PBs (▴), and sorted CD27^low^ PBs (●). PCs were defined as Annexin-V, 7-AAD, and CD20 negative, CD38 and CD138 positive.

Because absence of CD19 expression may be linked to PC longevity in vivo, we examined CD19 expression in PCs over extended in vitro cultures. Experiments performed using total unfractionated PBs or PBs sorted at day 6 into CD27^high^ and CD27^low^ populations demonstrated a consistent increase in the fraction of CD19^neg^ PCs over time (Fig. 6A). A rapid increase in the percentage of CD19^neg^ PCs generated by days 20–30 was followed by a gradual increase to day 100 in both experimental contexts as total PC number declined toward a plateau phase (Fig. 6B, 6C). Thus, CD19^neg^ PCs increase as a percentage of in vitro–generated LLPCs with extended culture.

### CD19^neg^ and CD19^pos^ PCs can be maintained independently

**FIGURE 7.**
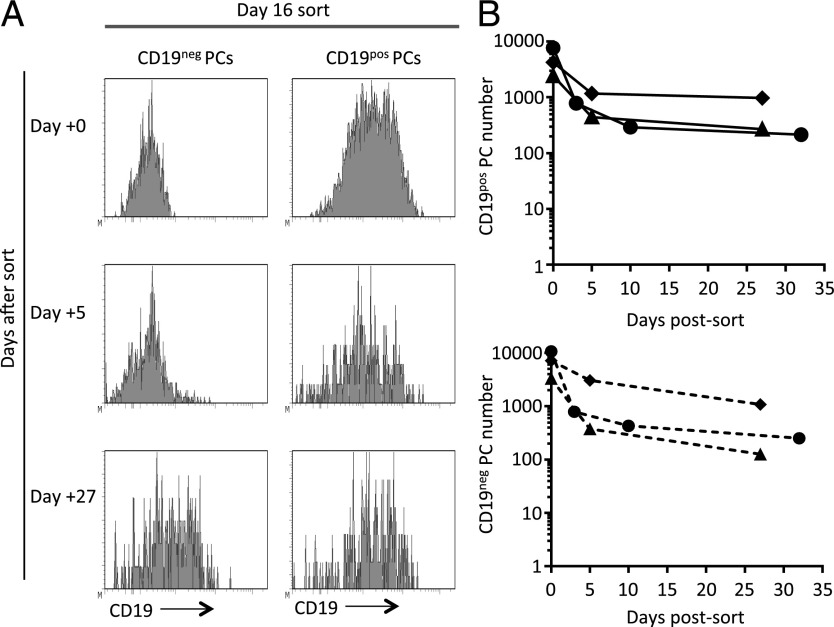
In vitro–generated CD19^neg^ PCs can be maintained independently of bulk CD19^pos^ PC fraction. PCs generated in vitro from peripheral blood B cells of healthy volunteers were sorted based on their CD19 expression on day 10 or 16 and maintained in vitro with conditions supporting PC survival. (**A**) Graphs showing changing levels of CD19 expression at the indicated days by PCs sorted on day 16 based on surface CD19. (**B**) The total numbers of CD19^pos^ (upper panel) and CD19^neg^ (lower panel) PCs maintained at the specified time points following flow sorting are shown. ●, donor 1 (day 10 sort); ▴, donor 2 (day 16 sort); ♦, donor 3 (day 16 sort).

The early establishment of CD19^neg^ PCs in vitro paralleled the early generation of CD19^neg^ ASCs in vivo. However, the continued presence of CD19^neg^ PCs over time in vitro could be consistent with preferential survival of CD19^neg^ PCs, or it could represent a subsequent age-associated transition in PC phenotypes with gradual loss of CD19 expression. To address these possibilities, we separated CD19^neg^ and CD19^pos^ PC fractions at day 10 or 16 of in vitro culture, achieving >99.6% purity of CD19^neg^ PCs, and seeded these along with the CD19^pos^ PC–enriched populations into extended in vitro cultures (Fig. 7A). For sorted CD19^neg^ and CD19^pos^ fractions, cell numbers initially declined postsort but then tended toward a plateau phase, as previously observed for total PCs in such cultures (Fig. 7B). Furthermore, within the enriched CD19^pos^ fraction, the percentage of CD19^pos^ cells remained stable over time, showing a <5% change at any time point. We conclude that CD19^neg^ PCs generated early during differentiation can be maintained independently of the bulk CD19^pos^ population in vitro for at least several weeks.

## Discussion

There is now increasing evidence in humans that LLPCs are enriched among the CD19^neg^ PC population within the BM and the small intestine (5, 16, 17). Therefore, the origins of such cells are of substantial interest. In this study, by examining the phenotype, function, Ag specificity, and *IGHV*-rearrangement repertoire of peripheral blood ASCs generated following seasonal influenza vaccination in healthy subjects, we provide evidence for the early emergence of Ag-specific CD19^neg^ ASCs during a human in vivo immune response.

It is not possible to track the relationship of these cells to the subsequent establishment of long-lived vaccine-specific PCs in the BM or elsewhere. These populations might plausibly still derive from CD19^pos^ PBs, which represent the bulk of PBs generated during the immune response, with age-associated loss of CD19 after seeding of an appropriate niche. However, our data provide an important proof of principle that CD19 loss can be established in a subset of PBs at an early stage of the immune response and, thus, at an early stage of the overall ASC life cycle. Interestingly, in recent data using carbon-14–based assessments of average PC age from human small intestine, of six donors tested, two were found to have CD19^neg^ PCs in the CD45^pos^ fraction with an average age indistinguishable from CD19^pos^ PCs and estimated as <1 y (5).

Although in vitro model systems cannot fully recapitulate the in vivo situation, they do provide the opportunity to address precursor/product relationships at a cellular level. In the context of such an in vitro model, our data indicate that CD19^neg^ PCs generated early during differentiation can persist in this state and can be maintained independently of the bulk CD19^pos^ PC population for at least several weeks. We are not able to track the fate of CD19^neg^ PBs generated early during differentiation in vivo. Thus, we cannot assess whether these directly or preferentially contribute to the LLPC population in BM or small intestine, nor can we conclude that CD19^pos^ PBs generated as the dominant element of the in vivo immune response do not contribute to establishment of the CD19^neg^ LLPC population through gradual loss of CD19 as the PCs age in an appropriate niche in vivo. However, we do consider that our data provide the basis for suggesting an alternate model: CD19^neg^ PBs/PCs generated early during an Ag-specific immune response may be recruited into supportive niche environments having lost CD19 expression before or just as they enter into the niche. If the CD19^neg^ PB/PC fraction generated early during an immune response were preferentially enriched for cells capable of seeding the LLPC compartment, this would have important implications for understanding the generation and maintenance of long-lived humoral immunity. However, we emphasize that, although we consider that our data open this possibility for future study, the results do not provide direct evidence to dispute the potential origin of CD19^neg^ LLPCs as a result of gradual age-associated loss of CD19 expression.

Our ability to detect influenza vaccine–specific CD19^neg^ PBs early during seasonal influenza vaccination responses contrasts with the results of Mei et al. (16), who reported a failure to detect Ag-specific CD19^neg^ PBs in the peripheral blood over a time course following tetanus toxoid vaccination. In their work, Mei et al. used intracellular flow cytometry for tetanus toxoid binding and cytoplasmic IgG staining to identify vaccine-specific ASCs. Intracellular staining provides a surrogate for secretory function and depends on detection of retained intracellular Ig following permeabilization, whereas ELISPOT provides a standard for detection of functional ASC populations. Therefore, it is possible that the explanation for the difference in results lies, in part, in the assays used, as well as the absolute number of events and volume of peripheral blood sampled to identify the rare CD19^neg^ PB fraction. Equally, the nature of the vaccinating Ag and the number of preceding vaccinations may impact on the frequency of CD19^neg^ PBs generated. In the case of influenza vaccination, the quality of the pre-existing immune response and donor vaccination history impact on the nature and magnitude of the detectable PB response, in particular when the same Ag is re-encountered (39–41).

In the steady-state, the peripheral blood PB population is thought to derive primarily from mucosal immune responses (42) and is enriched for IgA class-switched cells. In contrast, following a variety of vaccination approaches or during acute viral infection, dominant PB responses are generated, with kinetics generally peaking around days 6–7 postvaccination (35, 43, reviewed in Ref. 44). Such peripheral blood PBs are enriched for IgG-secreting cells and bear *IGHV* sequences with somatic hypermutations consistent with derivation from germinal center–experienced B cells (35, 45). The rapidity of their generation and high somatic hypermutation loads have been taken as evidence that such PBs derive from pre-existing memory B cells but not excluding the potential contribution of germinal center re-entry prior to re-emergence in the peripheral blood as a PB population with additional mutational diversity (35). Origin from a diverse memory B cell pool was also supported by longitudinal tracking of influenza vaccine–specific PBs over sequential years (41). The data presented in this article demonstrate that populations of CD19^neg^ PBs are present in the peripheral blood of healthy individuals in the absence of active infection or inflammation, as well as contributing to the expanded and proliferating Ag-specific immune response following vaccination. Thus, a capacity to generate functional Ag-specific CD19^neg^ ASCs is evident in vaccine responses of adult donors, and this capacity can be manifest in in vitro differentiations mimicking T-dependent or T-independent responses to the PC stage. Given the *IGHV* repertoire similarities that we observe, it is likely that, as for the dominant CD19^pos^ PB response, the CD19^neg^ PB population derives from pre-existing memory B cells in Ag-experienced donors.

The basis on which variable loss of CD19 is established during commitment to the PC state remains to be determined and might be influenced by the heritage of the differentiating B cell and the nature of the differentiation stimulus. The high degree of overlap between the in vivo PB fractions identified at the level of *IGHV* sequence strongly supports origin from a common precursor during the process of differentiation. The emergence of CD19^neg^ PB/PCs could represent more rapid progressive differentiation in one daughter cell relative to another, or it could be established in a deterministic or stochastic fashion among the differentiating population. There is considerable debate regarding the relative importance of deterministic and stochastic influences on decisions operating during the immune response (46–49). During PC differentiation, a central model links the process of cell division to stochastic choices of alternate fates, including class switching, differentiation, further division, or cell death in dividing activated B cell/PBs (46). It has been proposed that the timing of such potentially competing fates is determined at the initial point of cell activation/birth and may be influenced by subsequent exposure to stimuli within a family tree of differentiating lymphocytes (48). Thus, it is conceivable that the heterogeneity in CD19 expression among PBs sharing identical *IGHV* rearrangements, and thus presumed to belong to the same family tree, is established in a fashion related to this and might be explained mechanistically at the level of differential gene regulation. In this context, the heritage of the differentiating B cell and the nature of the instructive signal might be linked to the likelihood of related daughter cells establishing CD19 repression. Alternatively, in a progressive differentiation model, the cells most rapidly and completely losing CD19 expression would be more advanced in terms of differentiation. Although not excluding this explanation, our failure to observe CD19^pos^ PCs progressing to a CD19^neg^ state at a significant rate during in vitro culture after sorting argues against the view that, in the short-term, CD19^pos^ PB/PCs are lagging behind the CD19^neg^ PB/PCs in terms of the extent of differentiation. Therefore, in future work, it will be important to evaluate in more detail the factors influencing the establishment of the CD19^neg^ PCs generated early during differentiation. The data presented in this article provide proof-of-principle for the generation of vaccine Ag–specific CD19^neg^ ASCs early during human immune responses, indicating that heterogeneity in the extent of CD19 repression can be established during the initial transition of PBs to PCs and is not strictly dependent on PC ageing.

## Supplementary Material

Data Supplement
