# Supplementary material for: Early Emergence of CD19-Negative Human Antibody-Secreting Cells at the Plasmablast to Plasma Cell Transition
Source: J Immunol. 2017 May 10;198(12):4618–28. doi: 10.4049/jimmunol.1501761 (PMC5458329; doi:10.4049/jimmunol.1501761)
Supplement: Data Supplement [file JI_1501761.zip › JI_1501761_Supplemental_Material_1.pdf]

Supplemental Table 1

	Donor 1			Donor 2		
	CD19high	CD19low	CD19neg	CD19high	CD19low	CD19neg
Cells sequenced	10255	1821	1093	38935	7271	7515
Sequences	930120	643363	879608	1024866	1027333	1032207
Sequence depth per cell	83	322	723	25	132	132
Unique clones	3341	634	373	7928	2629	1775
Clones/input cell	0.33	0.35	0.34	0.20	0.36	0.24

	Donor 3			Donor 4		
	CD19high	CD19low	CD19neg	CD19high	CD19low	CD19neg
Cells sequenced	2870	469	224	11534	395	197
Sequences	1026002	1028930	1028932	1010449	1016384	1024741
Sequence depth per cell	287	1853	3861	80	2057	4446
Unique clones	1800	348	164	4892	277	122
Clones/input cell	0.63	0.74	0.73	0.42	0.70	0.62

Footnote: Supplemental Table 1-Summary of *IGHV* sequencing data. This table provides the numerical data for the number of cells sequenced, the total number of sequences obtained, read depths per cell, number of unique clones, and unique clones per input cell for each of the four donors, split according to the sorted PB fraction as shown in the table.

Supplemental Figure 1

(A)

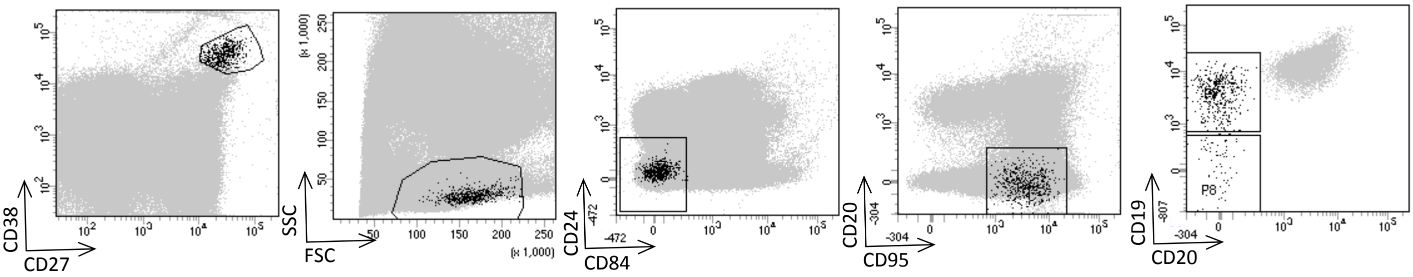

(B)

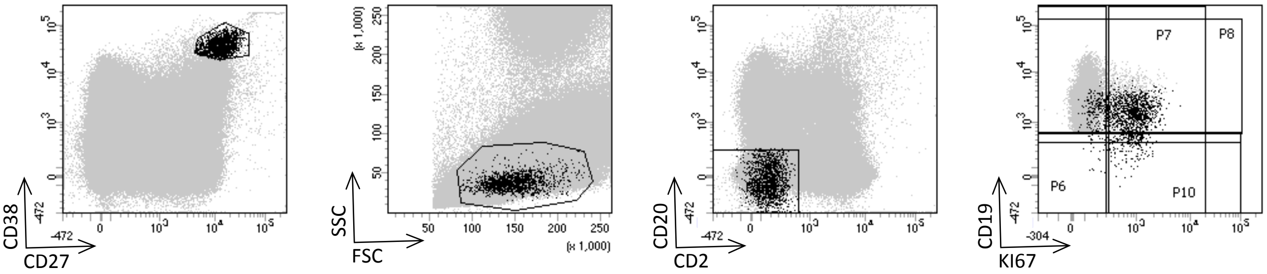

Supplemental Figure 1. Gating strategies for PB enumeration relating to Figure 3. (**A**) Show is the gating strategy used for enumeration of PB fractions in Figure 3A and 3B. The antigen used and sequence of gates is shown from left to right. Due to the low number of $CD19^{low}$ and $CD19^{neg}$ PB and the limited sample size available with repeated time course sampling, these fractions were considered together in this analysis. **(B)** Shown are the gating strategies used for the evaluation of Ki67 levels in PB fractions in Figure 3C. The sequence of gates is shown from left to right and the eventual gates used in identifying PB fractions for Ki67/CD19 expression on the far right.

Supplemental Figure 2

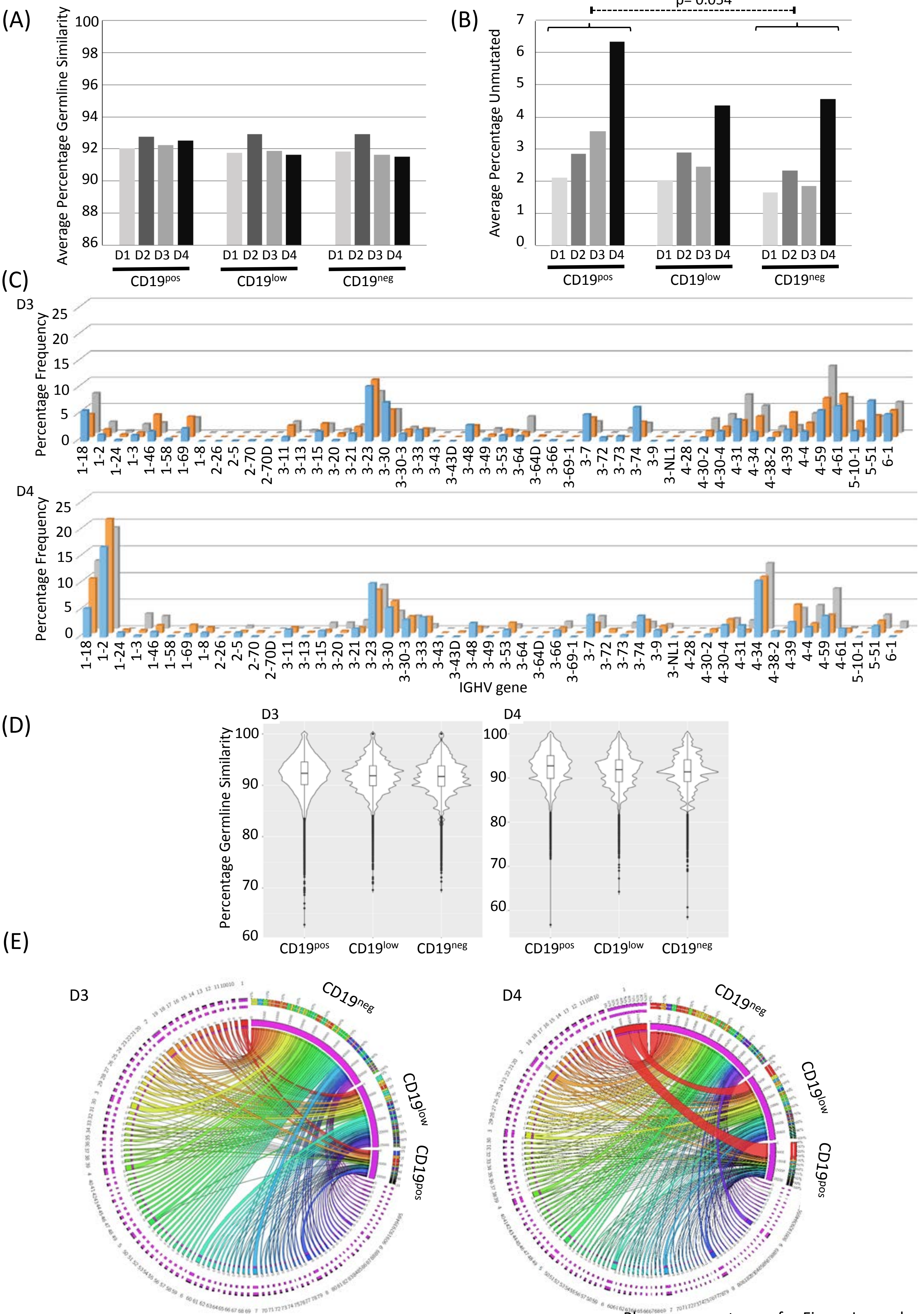

Please see next page for Figure Legend

Supplemental Figure 2. Gating strategies for PB enumeration relating to Figure 3. (**A**) Show is the gating strategy used for enumeration of PB fractions in Figure 3A and 3B. The antigen used and sequence of gates is shown from left to right. Due to the low number of CD19^{low} and CD19^{neg} PB and the limited sample size available with repeated time course sampling, these fractions were considered together in this analysis. **(B)** Shown are the gating strategies used for the evaluation of Ki67 levels in PB fractions in Figure 3C. The sequence of gates is shown from left to right and the eventual gates used in identifying PB fractions for Ki67/CD19 expression on the far right.

Supplemental Figure 3

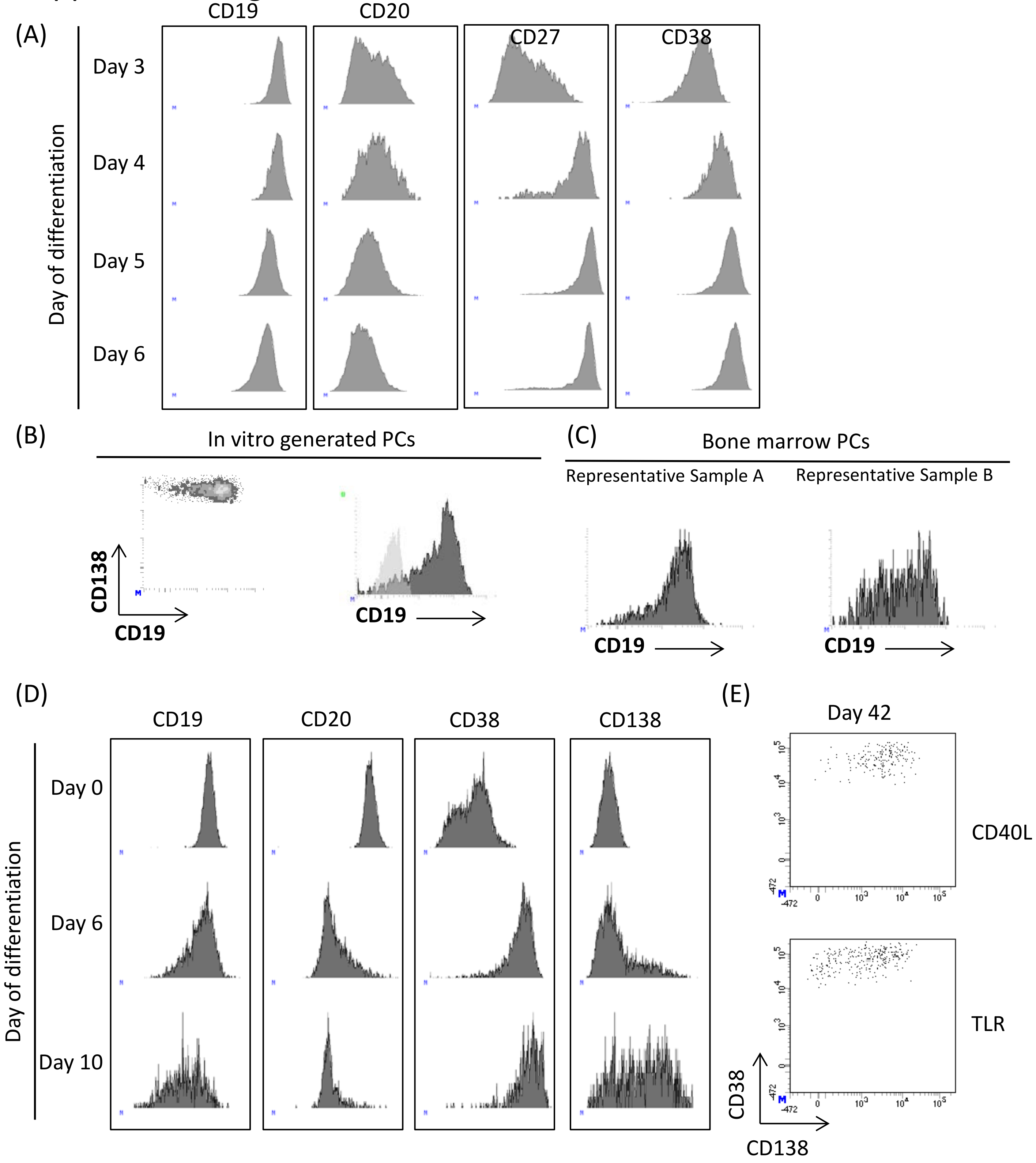

Supplemental Figure 3. Evaluation of CD19 expression on *in vitro* differentiating ASCs. Peripheral blood B-cells from healthy donors were exposed to T-D differentiation conditions (CD40L) and sampled at multiple time points for expression levels of B-lineage surface markers. (A) *In vitro* differentiated cells were analyzed during the transition from activated B-cell (day 3) to PB (day 6) for CD19, CD20, CD27 and CD38. (B) Cells obtained at day 10 using the *in vitro* culture system were assessed for co-expression of CD19 and CD138. Left panel shows CD19 vs CD138 expression, while right plot shows expression of CD19 (dark fill) relative to isotype control (light fill). (**C**) Representative patterns of CD19 surface expression from human bone marrow PCs for normal bone marrows from two representative donors (left and right panels labeled Sample A and Sample B), PCs were gated on CD27, CD38, and CD138. **(D)** Shows the sequence of antigen expression as in part (A) for *in vitro* differentiation using a TLR7/8 agonist based stimulation (right panels) compared to isotype controls (dashed line) on days 6 and 10. (**E**) Shows the phenotype of PCs for CD38 and CD138 expression at day 42 of differentiation for a representative donor using either a CD40L (upper panel) or TLR7/8 agonist (lower panel) base differentiation.
